# The Association of Neonatal Gut Microbiota Community State Types with Birth Weight

**DOI:** 10.3390/children11070770

**Published:** 2024-06-25

**Authors:** Wanling Chen, Kaiping Guo, Xunbin Huang, Xueli Zhang, Xiaoxia Li, Zimiao Chen, Yanli Wang, Zhangxing Wang, Rongtian Liu, Huixian Qiu, Mingbang Wang, Shujuan Zeng

**Affiliations:** 1 Shenzhen Clinical Medical College, Guangzhou University of Chinese Medicine, Shenzhen 518116, China; 2 Microbiome Therapy Center, South China Hospital, Medical School, Shenzhen University, Shenzhen 518111, China; 3 Division of Pediatrics, Longgang District Central Hospital of Shenzhen, Shenzhen 518116, China; 4 Division of Neonatology, Longgang District Central Hospital of Shenzhen, Shenzhen 518116, China; 5 Division of Neonatology, Shenzhen Longhua People’s Hospital, Shenzhen 518109, China; 6 Department of Burn Plastic Surgery, South China Hospital, Shenzhen University, Shenzhen 518111, China; 7 Department of Pediatrics, South China Hospital, Shenzhen University, Shenzhen 518111, China; 8 Department of Pediatrics, Shenzhen Second People’s Hospital, Shenzhen 518035, China; 9 Department of Neonatology, Longgang District Maternity & Child Healthcare Hospital of Shenzhen City (Longgang Maternity and Child Institute of Shantou University Medical College), Shenzhen 518172, China

**Keywords:** neonate, infant, low birth weight, gut microbiota, 16S rRNA gene sequencing, community state type, machine learning

## Abstract

Background: while most gut microbiota research has focused on term infants, the health outcomes of preterm infants are equally important. Very-low-birth-weight (VLBW) or extremely-low-birth-weight (ELBW) preterm infants have a unique gut microbiota structure, and probiotics have been reported to somewhat accelerate the maturation of the gut microbiota and reduce intestinal inflammation in very-low preterm infants, thereby improving their long-term outcomes. The aim of this study was to investigate the structure of gut microbiota in ELBW neonates to facilitate the early identification of different types of low-birth-weight (LBW) preterm infants. Methods: a total of 98 fecal samples from 39 low-birth-weight preterm infants were included in this study. Three groups were categorized according to different birth weights: ELBW (*n* = 39), VLBW (*n* = 39), and LBW (*n* = 20). The gut microbiota structure of neonates was obtained by 16S rRNA gene sequencing, and microbiome analysis was conducted. The community state type (CST) of the microbiota was predicted, and correlation analysis was conducted with clinical indicators. Differences in the gut microbiota composition among ELBW, VLBW, and LBW were compared. The value of gut microbiota composition in the diagnosis of extremely low birth weight was assessed via a random forest-machine learning approach. Results: we briefly analyzed the structure of the gut microbiota of preterm infants with low birth weight and found that the ELBW, VLBW, and LBW groups exhibited gut microbiota with heterogeneous compositions. Low-birth-weight preterm infants showed five CSTs dominated by *Enterococcus*, *Staphylococcus*, *Klebsiella*, *Streptococcus*, *Pseudescherichia*, and *Acinetobacter*. The birth weight and clinical indicators related to prematurity were associated with the CST. We found the composition of the gut microbiota was specific to the different types of low-birth-weight premature infants, namely, ELBW, VLBW, and LBW. The ELBW group exhibited significantly more of the potentially harmful intestinal bacteria *Acinetobacter* relative to the VLBW and LBW groups, as well as a significantly lower abundance of the intestinal probiotic *Bifidobacterium*. Based on the gut microbiota’s composition and its correlation with low weight, we constructed random forest model classifiers to distinguish ELBW and VLBW/LBW infants. The area under the curve of the classifiers constructed with *Enterococcus*, *Klebsiella*, and *Acinetobacter* was found to reach 0.836 by machine learning evaluation, suggesting that gut microbiota composition may be a potential biomarker for ELBW preterm infants. Conclusions: the gut bacteria of preterm infants showed a CST with *Enterococcus*, *Klebsiella*, and *Acinetobacter* as the dominant genera. ELBW preterm infants exhibit an increase in the abundance of potentially harmful bacteria in the gut and a decrease in beneficial bacteria. These potentially harmful bacteria may be potential biomarkers for ELBW preterm infants.

## 1. Introduction

Extremely-low-birth-weight (ELBW) infants refers to newborns whose birth weights are less than 1000 g. ELBW preterm infants have less well-developed systems than other low-birth-weight (LBW) preterm infants, and their poor immune systems make them more susceptible to infections and other preterm complications, often involving the nervous system, which increase the risk of cerebral palsy, intellectual disability, mission, and deafness [1,2]. The mortality rate of premature infants with ELBW is high. It was reported that the probability of ELBW premature infants dying from clinical complications (such as necrotizing enterocolitis and sepsis) is as high as 23% [3,4,5].

The gut microbiota begins to colonize the gastrointestinal tract at birth and plays an important role in the growth and development of newborns in the early stages of life and beyond. However, the diversity of gut microbiota is low in early neonatal life, and the structure of the gut microbiota is influenced by a variety of factors, including the mode of delivery, gestational age, birth weight, feeding method, and the environment [6,7,8,9,10,11]. The mode of delivery is one of the most important determinants of gut microbiota composition [12]. In vaginally delivered newborns, the abundance of *Bacteroidetes* is higher, while in cesarean-delivered newborns, *Klebsiella* and *Haemophilus* are the dominant species 6. Studies have shown that gestational age and birth weight are the most important factors influencing differences in intestinal microecology. Preterm infants have a unique gut microbiota in the early postnatal period [13], which is dominated by conditionally pathogenic bacteria, such as *Staphylococci*, *Enterococci*, and *Enterobacteria*, and beneficial bacteria such as *Bifidobacteria* do not exist as dominant species [14]. Most LBW preterm infants are transferred to a neonatal intensive care unit (NICU) after birth to be maintained on respiratory support equipment because of respiratory distress or other reasons. The gut microbiome colonization in LBW preterm infants can also be influenced by the NICU’s ambient settings and the usage of appropriate equipment. Extended respiratory support in preterm infants can lead to an increase in intestinal aerobic and facultative anaerobic bacteria [15]. Gut microbiota genera in LBW preterm infants in the NICU are dominated by *Klebsiella*, *Enterobacter*, and *Enterococci*, and differences among the gut microbiota decrease with an increase in hospitalization time [16]. An other significant element influencing the gut microbiota makeup in preterm newborns is feeding method. Breast-fed and non-breast-fed infants have different gut microbiota [17]. However, breastfeeding can help premature infants’ immune systems mature and encourage the colonization of intestinal bacteria *Bifidobacterium* [18]. The maternal diet can also affect the composition of the infant’s gut microbiota [19,20,21,22]. For instance, if the mother consumes plant-based protein or a high-fat diet, it can lead to a significant reduction in the presence of *Bacteroides* bacteria in the newborn’s gut, and the decrease in *Bacteroides* may affect the early-immune and metabolic development of newborns [20,21]. In addition, the use of antibiotics also has a certain impact on the composition of gut microbiota in premature infants. Antibiotics can reduce the diversity of gut microbiota and delay the colonization of *Bifidobacterium* [23]. The community state type (CST) is based on the gut microbiota abundance obtained from sequencing analysis and classified into different CSTs by clustering [24,25]. There are also variations in the types of gut microbiota-community states among infants of different age groups. In healthy infants under 6 months old, the gut microbiota CSTs are mainly characterized by a higher abundance of *Bifidobacterium*, while in infants aged 12 to 36 months typical adult bacterial genera such as *Bacteroides* and *Faecalibacterium* predominate [26]. It can be seen that, as the newborn grows and develops, the composition of gut microbiota in the body also undergoes dynamic changes.

Current research has found that preterm infants, because of their prolonged exposure to the NICU environment and the relatively frequent clinical interventions such as respiratory support and antibiotic use they experience, undergo changes in their gut microbiota composition, making them more susceptible to conditions like NEC and late-onset sepsis (LOS) [3,27,28]. Supplementing the food of early-stage newborns with probiotics such as *Bifidobacterium* can promote the colonization of the intestine by beneficial bacteria, thereby preventing or reducing the occurrence of NEC, LOS, and feeding intolerance [29,30,31,32]. Probiotic supplementation improves gut microbial composition, making it closer to that of full-term infants, which is beneficial for promoting immunity and metabolism [33,34,35,36]. Probiotic-supplemented ELBW preterm newborns had low levels of harmful bacteria and a substantial increase in the gut bacterial *Bifidobacterium*. The results showed that the abundance of *Bifidobacterium* in the intestinal bacteria of preterm infants of ELBW who received probiotic supplementation was significantly higher than those who did not receive probiotics, and the abundance of pathogenic bacteria was lower. Simultaneously, preterm infants who received probiotic supplementation had higher levels of acetate and lactate (end products of HMO metabolism), and the abundance of acetate was positively correlated with the abundance of *Bifidobacterium* [37]. At the same time, the gut microbiota diversity of ELBW preterm infants who received probiotic *Lactobacillus* supplementation increased, and the abundance of the supplemented probiotics also rose. Compared with the control-group infants, ELBW preterm infants who received probiotic supplementation had reduced abundances of *Staphylococcaceae* and *Enterobacteriaceae* in their intestines [38]. It can be seen that probiotic supplementation for preterm infants can facilitate colonization of the intestine by beneficial bacteria and reduce harmful bacteria. Probiotics can also promote the metabolism of HMO in breast milk, enabling the beneficial metabolites in HMO to exert their immune-enhancing effects.

Most studies on intestinal microbiota have focused on full-term infants; however, the health outcomes of ELBW and VLBW preterm infants are equally important. Because of their immature systemic physiology and immature intestinal microbiota structure, they may be predisposed to long-term outcomes such as neurodevelopmental disorders [39]. Studies have found that there is a correlation between gut microbiota and brain function. A study established a connection between the gut microbiota, immunology, and neurodevelopment in extremely-preterm infants and discovered that excessive growth of the intestinal microbiota can be a strong predictor of brain injury. Abnormal development of the gut-microbiota–immune-system–brain axis may drive or exacerbate brain injury in extremely-preterm infants [40]. The underlying mechanisms of these effects have not been fully elucidated, and some have not even been considered. Therefore, this study aimed to investigate the gut microbiota structure of preterm infants with LBW using 16S rRNA gene sequencing technology. We analyzed the gut microbiota structure, corresponding microbiota profiles, and the CST of the gut microbiota among preterm infants of different birth weights. Correlation analysis of the CST and clinical indicators of preterm infants was conducted, and the clinical value of the intestinal microbiota in diagnosing extremely-LBW preterm infants was evaluated.

## 2. Methods

### 2.1. Participant Enrollment and Sample Collection

This study included a total of 98 fecal samples from 39 preterm infants with LBW. Inclusion criteria: premature infants hospitalized in the NICU of the neonatology department; gestational age at birth of <37 weeks and a birth weight of <2500 g; hospitalization time > 7 days. Exclusion criteria: neonates with a gestational age at birth of ≥37 weeks and a birth weight of ≥2500 g; hospitalization time < 7 days; premature infants with severe congenital heart disease and severe digestive tract malformation who need surgery; premature infants with Down syndrome, hereditary metabolic diseases and severe asphyxia; stillbirths, induced abortions, combined with severe cardiac and renal dysfunction. We selected the first stool sample of NICU low-birth-weight premature infants who met the inclusion criteria, then planned to collect fecal samples every 2 weeks until discharge or until the 8th week of collection. Finally, the preterm infants were divided into three groups based on their birth weights: ELBW (<1000 g), VLBW (1000–1499 g), and LBW (1500–2499 g).

The guardians of the participants collected fecal samples in sterile containers and transported them overnight on ice to the laboratory. The researchers immediately aliquoted the samples into tubes containing 3–5 g each and stored them in a −80 °C freezer. The research protocol of this study was approved by the hospital’s medical ethics committee, and each neonate’s parents provided written informed consent. The research protocol was designed in compliance with the Helsinki Declaration and approved by the hospital’s medical ethics committee.

### 2.2. Analysis of Gut Microbiota

Refer to our published articles [41,42,43,44,45,46] for detailed methodology on 16S rRNA gene sequencing and bioinformatic analyses (detailed in the Supplementary Materials).

### 2.3. Analysis of Ecological Diversity Indices

The diversity function from the R package Vegan (version 2.6-4) was used to calculate the Shannon and Inverse Simpson indices for the samples. The estimateR function from the R package Vegan was used to calculate the richness index for the samples.

### 2.4. Stacked Bar Chart, Chord Diagram, Venn Plot, Volcano Plot, Manhattan Plot

The processes used to obtain the stacked bar charts, chord diagrams, Venn plots, volcano plots, and Manhattan plots were completed by referring to the EasyAmplicon protocol [47].

### 2.5. Constrained Principal Coordinates Analysis

Constrained Principal Coordinate Analysis (CPCoA) refers to the addition of grouping information to the Principal Coordinate Analysis (PCoA) in order to find a plane that can best explain the differences between groups under self-defined grouping conditions. The process was completed by referring to the EASYAMPLICON protocol [47].

### 2.6. Gut-Microbiota Network Analysis

The layout and visualization of the gut microbiota network diagram were completed with reference to the article published by the Zhou Jizhong Team, Li Ji Team, and Shen Qirong Team [48,49,50].

### 2.7. Analysis of Microbial Community Structure

Gap statistics were used to determine the optimal number of clusters in the microbial community structure. This method identifies the best number of clusters by comparing the distribution of clustered data with that of a random distribution through the calculation of the gap (or “gap statistic”) between them.

### 2.8. Non-Metric Multidimensional Scaling

Non-metric multidimensional scaling (NMDS) was completed with reference to the authors’ previously published research [51,52]. Firstly, based on the genus-level data, the metaMDS function in the R package Vegan (version 2.6-4) was used to conduct NMDS ordination analysis and obtain the stress value. Simultaneously, the adonis2 function in the R package Vegan was employed to conduct a permutational multivariate analysis of variance (PERMANOVA) based on Bray–Curtis distance, yielding *p*-values and R^2^ values. The ordisurf function in the R package Vegan was used to passively add environmental variables to the NMDS ordination. Finally, the geom_point function in the R package ggplot2 (version 3.3.2) was employed to visualize the results of the NMDS ordination.

### 2.9. Random Forest Analysis

Refer to previously published articles [53,54,55] for detailed methodology on the random forest analysis (detailed in the Supplementary Materials).

### 2.10. Other Analyses

To evaluate the correlation between the significantly different gut microbiota compositions between groups and clinical manifestations, the lm function in R software (version 4.2.3) was used to construct a logistic regression model. The *p*-value and coefficient of determination (R-squared) of the logistic regression model were obtained through the summary function. The beeswarm function in the R package beeswarm (version 0.4.0) was used to create boxplots, and the wilcox.test function from the R package stats (version 4.2.3) was used for statistical testing to obtain *p*-values. The visualization of clinical data and other aspects were completed using customized scripts.

## 3. Results

**Figure 1:**
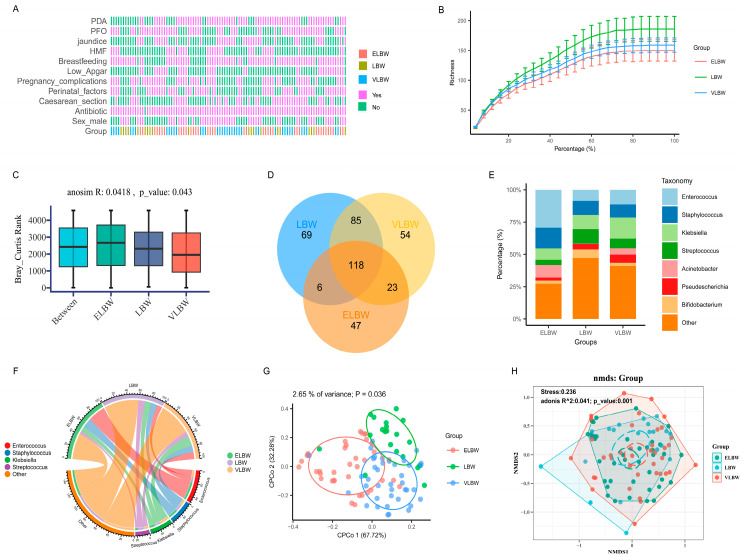
Analysis of gut microbiota diversity in low-birth-weight preterm infants. (**A**) Visual analysis of clinical data for three groups of preterm infants; (**B**) saturation curve analysis based on species richness; (**C**) comparison of similarities and differences in ELBW, LBW, and VLBW gut microbiota composition data by ANOSIM; (**D**) Venn plot illustrating shared and unique OTUs among the three groups; (**E**,**F**) composition of gut microbiota at the genus level among the three groups; (**G**) CPCoA explained 2.65% of the total variation in gut microbiota composition among the groups, and there were significant differences between the groups (*p* < 0.05); (**H**) NMDS analysis used to rank the gut microbiota of the ELBW, VLBW, and LBW groups. The Bray–Curtis index was calculated for the three groups to generate the NMDS to visualize the similarities among the gut microbiota, and the results showed that there was a significant difference in the distribution of the gut microbiota among the three groups (*p* < 0.05).

This study included 98 fecal samples from 39 preterm infants. We conducted a visual analysis of clinical data on premature infants, and the results are shown in Figure 1A. To determine the saturation of sequencing data for the 16s rRNA gene, that is, whether the number of sequencing data were sufficient, we performed saturation curve analysis based on species richness, and the results are shown in Figure 1B. It can be seen that the saturation curves for ELBW, LBW, and VLBW all tended to saturate, indicating that the 16s rRNA gene sequencing data were sufficient. At the same time, the species richness in the LBW group was slightly higher than those of ELBW and VLBW infants. We used ANOSIM, which stands for analysis of similarities, to compare the similarity of the gut microbiota composition data among ELBW, LBW, and VLBW infants. As a non-parametric test method, ANOSIM is often used to test for the similarities among high-dimensional data. We also compared the magnitude of differences in gut microbiota compositions both between and within the groups of ELBW, LBW, and VLBW infants, and the results are presented in Figure 1C. The R-value of 0.0418 indicated the presence of a certain degree of difference both within and between the groups. The *p*-value of 0.043 suggested that this difference was restricted. To further understand the shared and unique gut microbiota profiles among ELBW, LBW, and VLBW infants, and to visually demonstrate the overlaps in gut microbiota among the three groups, we conducted an analysis using a Venn plot and found that the number of OTUs shared among the three groups was 118, indicating that the majority of gut microbiota were common to ELBW, LBW, and VLBW infants (Figure 1D).

To further understand the gut microbiota composition of preterm infants, we analyzed the gut microbiota at the genus level (Figure 1E,F). The results showed that the gut microbiota of the ELBW group was dominated by *Enterococcus*, followed by *Staphylococcus*, *Acinetobacter*, and *Klebsiella*. The gut microbiota of the VLBW group was primarily composed of *Klebsiella*, followed by *Enterococcus*, *Staphylococcus*, *Streptococcus*, *Acinetobacter*, and *Pseudescherichia*. In the LBW group, *Enterococcus*, *Staphylococcus*, *Klebsiella*, and *Streptococcus* were the main gut microbiota genera, followed by *Bifidobacterium* and *Pseudescherichia*. Compared with those of the LBW group, the ELBW and VLBW groups’ abundances of *Acinetobacter* were significantly increased, with a notable increase observed in the ELBW group. Conversely, the abundance of *Bifidobacterium* was significantly reduced. We employed CPCoA to compare the differences in the gut microbiota composition among the ELBW, LBW, and VLBW groups of infants. The results showed that the grouping could explain 2.65% of the variation, and the separation was relatively distinct, indicating that grouping had a certain influence on the composition of gut microbiota (Figure 1G). We further analyzed the gut microbiota of preterm infants in the ELBW, LBW, and VLBW groups by NMDS clustering at the genus level. The group data were calculated via the Bray–Curtis index to generate NMDS to visualize the similarity of the gut microbiota. In Figure 1H, each point in the graph represents the microbiota characteristics of an individual preterm infant in a low-dimensional space. The results showed that there were distinct clusters of gut microbiota genera among the three groups, indicating significant differences in their distribution (R^2^ = 0.041, *p* = 0.001). Simultaneously, we conducted clustering analysis based on the clinical phenotypes of the three groups of preterm infants. The results showed significant differences in gestational age and birth weight among the three groups (Figure S1A).

**Figure 2:**
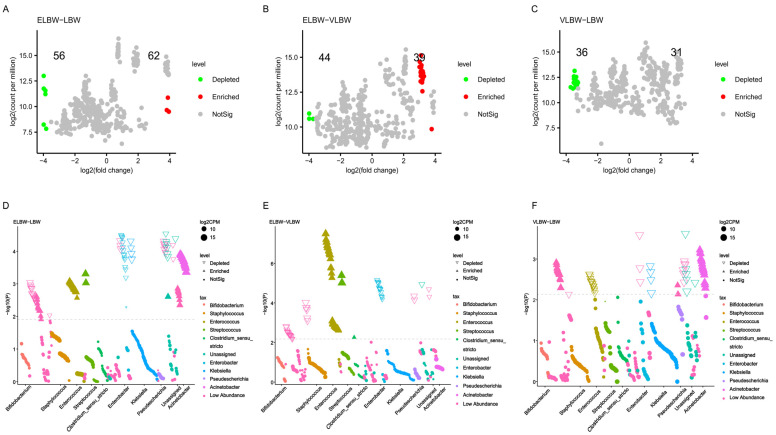
The distribution of bacteria at the genus level in the gut microbiota. (**A**–**C**) Volcano plots revealing differentially abundant gut microbiota among the VLBW, ELBW, and LBW groups. Red represents significantly high-abundance bacteria, while green represents significantly low-abundance bacteria. (**D**–**F**) Manhattan plots revealing the distribution of gut microbiota among the three groups.

To further understand whether the gut microbiota components were differentially distributed among the ELBW, LBW, and VLBW groups, we performed an analysis of gut microbiota at the genus level by volcano plots. The results showed that 118 genera with differential abundances were identified between ELBW and LBW at the genus level. Among them, 56 genera were less abundant in ELBW, while 62 genera were more abundant in the ELBW than in the LBW group (Figure 2A). Compared with VLBW infants, ELBW infants exhibited a total of 83 differentially abundant genera of gut microbiota at the genus level, with 44 genera showing lower, and 39 genera showing higher, abundances compared with those in the VLBW group (Figure 2B). A total of 67 differentially abundant genus-level enterobacteria were identified in VLBW infants compared with the findings in LBW infants, with 36 genera less abundant and 31 genera more abundant than in the LBW group (Figure 2C). We further specifically analyzed these differentially abundant gut microbiota through Manhattan plots. The results showed that, compared with the LBW group, the ELBW group exhibited more *Enterococcus*, *Streptococcus*, and *Acinetobacter*, but lower amounts of *Klebsiella*. *Enterococcus*, *Streptococcus*, and *Clostridium sensu stricto* abundances were predominantly lower, and that of *Enterobacter* was predominantly higher, in ELBW compared with the findings in VLBW. Compared with LBW infants, VLBW infants showed more *Acinetobacter* and less *Enterococcus* and *Klebsiella* (Figure 2D–F).

**Figure 3:**
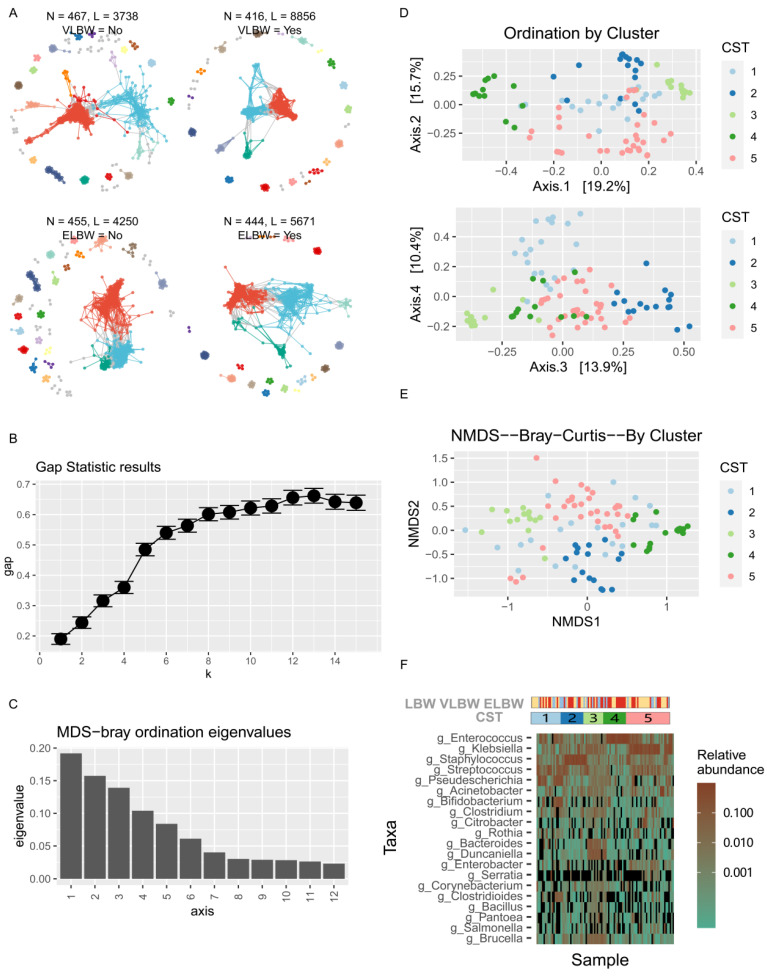
The relationships among the five clusters of samples identified through clustering analysis and the diversity of the gut microbiota. (**A**) MENs method based on 16S rRNA high-throughput sequencing and visualization tools to analyze the interrelationships among gut microorganisms between groups. (**B**) The gap statistic method was used to analyze the optimal number of clusters based on the Bray–Curtis distance of the incoming samples; the results show that 5 was the optimal k value. (**C**) Ordination analysis of eigenvalue obtained from MDS. (**D**) NMDS visualization based on the first four eigenvectors obtained by MDS. (**E**) Demonstration of 5 CSTs samples based on the NMDS method. (**F**) Heatmap showcasing the variations in the abundance of driver intestinal bacteria across the five CST sample.

To further understand the interrelationships among the intestinal microbiota in each group, we employed the molecular ecological networks (MENs) method and visualization tools based on 16S rRNA high-throughput sequencing. The results showed that the gut microbial interaction network of the VLBW group consisted of 416 nodes (ASVs) and 8856 links (interactions). In the network constructed for the ELBW group, more nodes were observed, but fewer links were present (Figure 3A). Compared with non-breastfed preterm infants, breastfed preterm infants exhibited a higher number of nodes but fewer links in their gut microbiota networks. Preterm infants with jaundice had fewer nodes and even fewer links compared with those without jaundice (Figure S1B). To further understand whether the differences between all enrolled subjects affected their corresponding gut microbiota and clinical phenotypes, for example, we used gap statistics, a clustering method based on interval statistics, and analyzed the optimal number of clusters based on the total sample size. The study subjects were grouped according to their similarities, resulting in high similarity levels within groups and significant differences between groups. Figure 3B displays the gap statistic plots based on clustering by sample size. Based on B = 100 iterations for each k, the results showed that k = 5 was the optimal k-value, indicating that the clustering performance was basically optimal. As k continued to increase, the performance improved, relatively slowly. Therefore, the final clustering algorithm was chosen with a k-value of 5, meaning that we grouped the samples into five clusters. We further employed NMDS to analyze the five clusters identified through the clustering analysis. By calculating the Bray–Curtis index, we generated an NMDS plot to visually display the similarities among the samples. To further understand the connection between the gut microbiota and clinical phenotypes in preterm infants with low birth weight, we first conducted an analysis of CSTs based on their gut microbiota. Through multidimensional scaling (MDS), we performed ordination analysis based on the sorting of eigenvalues and visualized the first four eigenvectors using NMDS (Figure 3C,D). Then, five CST samples were visualized using the NMDS method. In Figure 3E, each point on the plot represents the characteristics of a single sample in the low-dimensional space, and the results indicated that the five CSTs exhibited distinct clustering patterns. To understand the relationships among the gut microbiotic abundances of the five clusters identified through clustering analysis, we used a further clustering heatmap to display the variations in the abundance of key gut microbiota across the five CST samples. This allowed us to compare the compositional similarities and differences in the gut microbiota at the genus level among the different groups. The results indicated that the gut microbiota in the five CSTs was primarily composed of harmful bacteria. The six bacteria species with relatively high abundance in the gut microbiota were *Enterococcus*, *Klebsiella*, *Staphylococcus*, *Streptococcus*, *Pseudescherichia*, and *Acinetobacter*. The abundance of gut bacteria also varied among the different CSTs. Specifically, the abundances of *Streptococcus* and *Pseudescherichia* were higher in CST 1; *Staphylococcus* had a higher abundance in CST 2, *Enterococcus* was more abundant in CST 4, and *Klebsiella* was more prevalent in CST 5 (Figure 3F).

**Figure 4:**
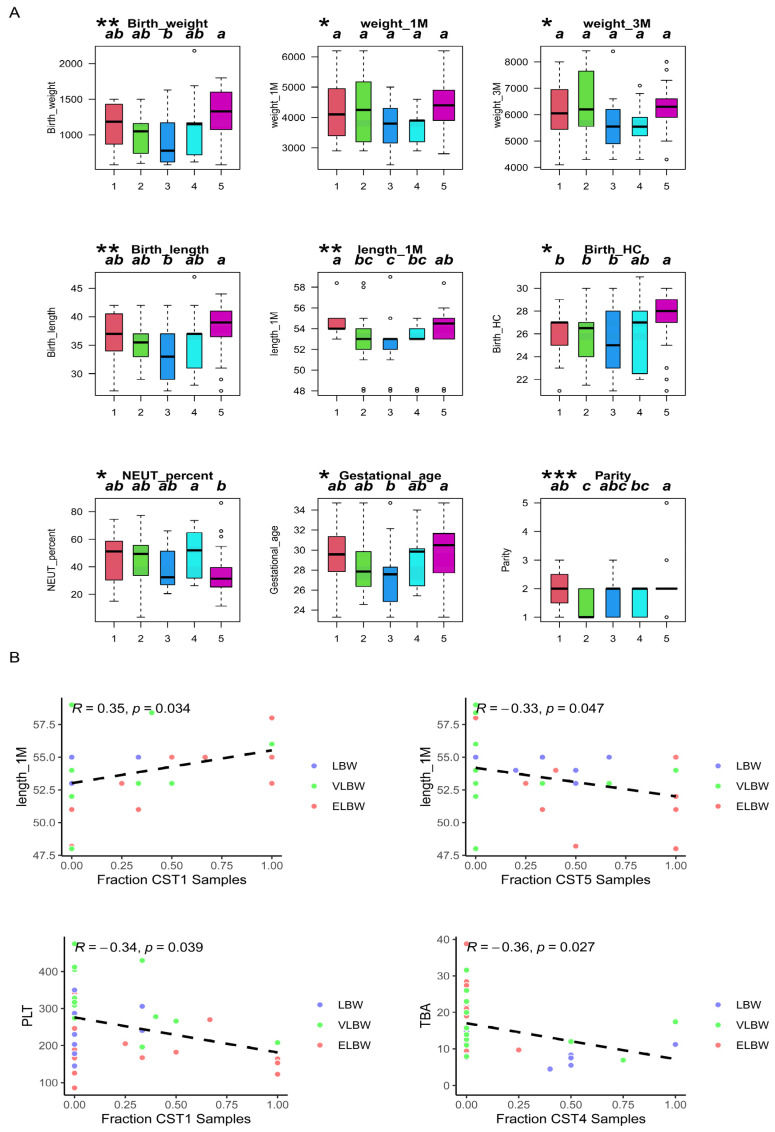
The correlation analysis between the five CST samples and clinical phenotypes. (**A**) Significant differences between the five CST samples and clinical phenotypes (*** *p* < 0.001, ** *p* < 0.01, * *p* < 0.05); a, b, and c are defined as using the significant difference letter marking method to arrange all the means from largest to smallest. Any difference with the same marking letter is not significant, and any difference with a different marking letter is significant. (**B**) Significant linear correlation between the five CST samples and clinical phenotypes.

We further analyzed the relationships among the five CSTs and clinical phenotypes, and the results are presented in Figure 4A. Overall, there were significant differences (*p* < 0.05) between the five CSTs in terms of gestational age, parity, birth weight and length, weight and length at 1 month, weight and length at 3 months, and the percentage of neutrophils. In terms of the gestational age, birth weight, and birth length of the infants, there were significant differences between CST 3 and CST 5. In a comparison of body length at 1 month, there were significant differences between CST 1 and CST 3. In a comparison of body length at 3 months, CST 5 exhibited significant differences compared with CST 1, CST 2, and CST 3. There were also significant differences in the percentage of neutrophils between CST 4 and CST 5. In terms of parity, there was also a significant difference between CST 2 and CST 5. We further analyzed the correlation between each group and the clinical indicators (Figure 4B). The results showed a significant positive correlation between body length at 1 month and CST 1, while there was a significant negative correlation between body length at 1 month and CST 5. Additionally, there was a significant negative correlation between CST 1 and platelet count (PLT), as well as a negative correlation between CST 4 and total bile acid (TBA). 

**Figure 5:**
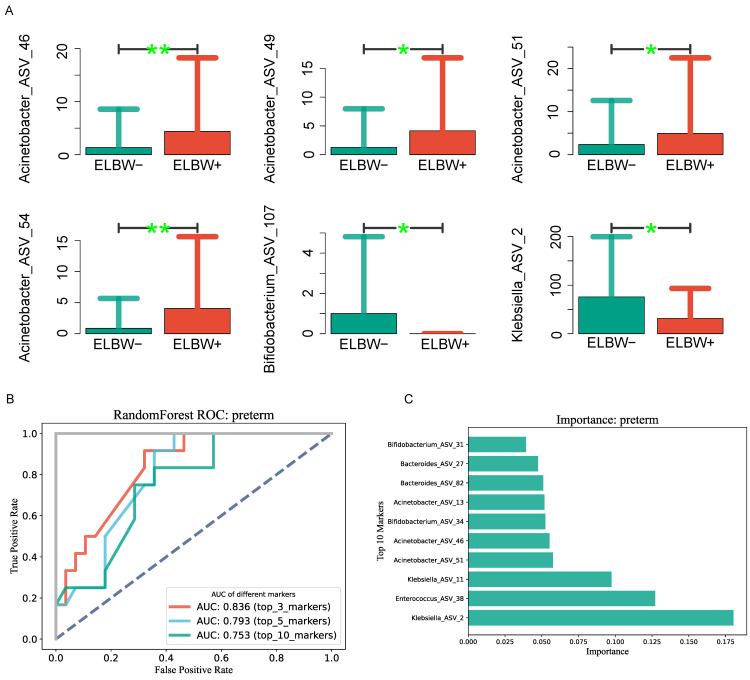
Intestinal bacteria may be a potential biomarkers for ELBW premature infants. (**A**) The abundances of intestinal bacteria *Acinetobacter*_ASV_46, *Acinetobacter*_ASV_49, *Acinetobacter*_ASV_51, and *Acinetobacter*_ASV_54 were significantly elevated in the ELBW infant group. The abundances of intestinal bacteria *Bifidobacterium*_ASV_107 and *Klebsiella*_ASV_2 were significantly reduced in the ELBW group of infants. ** *p* < 0.01, * *p* < 0.05. (**B**,**C**) Intestinal bacteria can be used as potential biomarkers for ELBW preterm infants. (**B**) Based on the random forest model, the potential use of intestinal bacteria in the classification of ELBW preterm infants was evaluated. The results showed that the AUC value of the top-three intestinal bacteria in the classification of ELBW preterm infants was 0.836. (**C**) Rank of intestinal bacterial markers.

To further explore whether there were differences in the gut microbiota between ELBW infants and other LBW infants, we initially classified the preterm infants into two groups. One was the ELBW group (ELBW+), and the other group comprised infants with VLBW and LBW, collectively known as the non-extremely-low-birth-weight group (ELBW−). According to the classification of gut microbiota under ASV conditions, the gut microbiota of the two groups of children were compared, and the results are shown in Figure 5A. The four bacteria with significantly increased abundance in the ELBS+ group were *Acinetobacter*_ASV_46, *Acinetobacter*_ASV_49, *Acinetobacter*_ASV_51, and *Acinetobacter*_ASV_54. The abundance of intestinal bacteria *Bifidobacterium*_ASV_107 and *Klebsiella*_ASV_2 were significantly lower in the ELBS+ group of infants. To further evaluate the clinical application value of the gut microbiota, we constructed a classifier based on a random forest model, as shown in Figure 5B,C. The top-three gut microbiota (*Klebsiella*_ASV_2, *Enterococcus*_ASV_38, *Klebsiella*_ASV_11) used for preterm infant classification had an AUC value of 0.836. The AUC values for preterm infant classification using the top-5 and top-10 gut microbiota were 0.793 and 0.753, respectively. These results indicated that intestinal bacteria may be potential biomarkers for ELBW preterm infants.

## 4. Discussion

The CSTs can be used to discover the dominant bacterial community composition in different age groups and samples. Currently, most studies have focused on analyzing the CSTs of gut microbiota based on samples from the reproductive tract. One study categorized the female vaginal microbiota into five CSTs by 16S rRNA gene sequencing, of which CSTs I, II, III, and V were all dominated by *Lactobacillus* 24. A study based on adult gut microorganisms found that adult gut microorganisms can be categorized into three distinct clusters, known as enterotypes, driven by different genera of bacteria, namely *Bacteroides* (enterotype 1), *Prevotella* (enterotype 2), and *Ruminococcus* (enterotype 3) [56]. A study conducted on the gut microbiota of school-age children identified three distinct enterotypes: *Bacteroides*, *Prevotella*, and *Bifidobacterium* [57].

There have been fewer CST analyses conducted on samples from neonates. A study focusing on the gut microbiota of infants found that infants under 6 months of age primarily had five community state types, which were dominated by the genus *Bifidobacterium*. There were seven main infant community state types (ICSTs) for infants aged 6–36 months; these ICSTs were characterized by typical adult bacterial genera and primarily manifested as decreased *Bifidobacterium* and increased *Bacteroides* 24. Grier and his team conducted a longitudinal CST analysis by collecting intestinal samples from preterm and full-term infants. The results revealed the existence of CSTs potentially characterized by *Enterobacteriaceae*, *Veillonella*, *Ruminococcus*, *Streptococcus*, *Prevotella*, *Bacteroides*, and *Bifidobacterium* [58]. The detection of a large number of ICSTs is believed to reflect the high variability and dynamics of the microbiota during early life [59]. In this study, CST analysis was conducted on the gut microbiota of low-weight preterm infants, and we found diverse gut microbiota compositions among the VLBW, LBW, and WELBW infants. Low-weight preterm infants exhibited five distinct CSTs, primarily characterized by *Enterococcus*, *Staphylococcus*, *Klebsiella*, *Streptococcus*, *Pseudescherichia,* and *Acinetobacter*. The primary intestinal bacteria in CST 1 were *Streptococcus* and *Pseudescherichia*. CST 2 was dominated by *Staphylococcus*. CST 4 was primarily made up of *Enterococcus*, while CST 3 and CST 5 were mainly *Klebsiella*. It can be seen that the CSTs of the neonatal intestine were generally dominated by opportunistic pathogens.

The gut microbiota of neonates is influenced by various factors, and there is a correlation between the community state types of neonatal microbiota and clinical phenotypes. The community state types of the neonatal gut microbiota also differ based on the mode of delivery. Infants delivered vaginally tend to have CSTs dominated by *Bifidobacterium*, while those delivered by cesarean section are more likely to have *Bacteroides* as the primary bacteria 24. In this study, preterm infants exhibited significant differences in gestational age, birth weight, and birth length in terms of CSTs, and there were especially significant differences between CST 3 and CST 5. There was a linear relationship between the CST and the length, PLT, and TBA of preterm infants. However, further verification is needed to determine whether there is a causal relationship between the gut microbiota and these clinical indicators. 

Particularly in preterm children, the degree of intestinal growth is immature during the neonatal era, and the gut microbiota’s structure and function varies markedly. The gut microbiota of preterm infants is often dominated by facultative anaerobic and opportunistic pathogens such as *Enterobacter*, *Enterococcus*, and *Staphylococcus* [60,61]. In this study, we analyzed the structure of the intestinal bacteria in different low-birth-weight preterm infants. We discovered that, although the intestinal bacterial composition of preterm infants with different low-birth-weights varied, the main bacterial species were still opportunistic pathogens such as *Enterococcus*, *Staphylococcus*, *Klebsiella*, *Streptococcus*, and *Acinetobacter*. Compared with the VLBW and LBW groups, the ELBW group in this study exhibited a significant increase in the potentially harmful intestinal bacterial genus *Acinetobacter*. *Acinetobacter* belongs to the category of opportunistic pathogens, is also a major cause of neonatal infections and outbreaks in neonatal intensive care units (NICUs) [62], and can lead to the occurrence of diseases such as meningitis, bloodstream infections, and respiratory infections [63,64]. *Acinetobacter*, one of the major drug-resistance-associated mortality pathogens, is associated with high morbidity and mortality rates, and preterm and VLBW infants are highly susceptible to infection [65,66].

In this study, *Klebsiella* was identified as a potential biomarker bacteria genus in preterm infants. The random forest analysis also indicated that *Klebsiella* could be a potential biomarker for diagnosing preterm infants. *Klebsiella* is a common intestinal microorganism during neonatal development [67]. It can act on macrophages to thereby evade the host immune system and to persist, potentially causing opportunistic infections [68]. Because of their gestational age and low body weight, premature infants do not yet possess fully developed or matured systems such as digestion and absorption and immune systems. Preterm infants are more prone to a series of infections, such as neonatal sepsis and necrotizing enterocolitis (NEC), because of their small gestational age, low body weight, and incomplete development of various systems, such as the digestive, absorption, and immune systems. Relevant studies have shown that *Klebsiella* is associated with bacterial infections and the occurrence of NEC in neonates [69,70], and elevated *Klebsiella* abundance is also associated with neonatal cerebral white-matter damage 40. However, further research is needed to elucidate the specific mechanisms underlying these associations and their relevance to the health of preterm infants.

In this study, a significant decrease in the abundance of the intestinal probiotic *Bifidobacterium* was observed in ELBW preterm infants. *Bifidobacterium* are beneficial bacteria in the human gut with pro-inflammatory, anti-inflammatory, anti-viral, and immunomodulatory functions [71,72,73]. Studies have found that a higher abundance of *Bifidobacterium* in early infancy is associated with a better immune response to vaccination and potentially enhanced immune memory [74]. A low abundance of *Bifidobacterium* may lead to the development of allergies, eczema, and asthma [75]. A study of gut microbial compositions and functions in very-preterm infants given probiotics found that *Bifidobacterium* can be used to predict microbial maturation and that *Bifidobacterium* is an important factor in accelerating gut microbial maturation 35. It showed that probiotic supplementation can promote the maturity of gut microbiota in premature infants, thus reducing differences between microbiota. In addition, related studies found that probiotic supplementation in preterm infants can reduce mortality and improve NEC and feeding intolerance, among other benefits [76]. Evidently, changes in intestinal probiotics may affect the health of preterm infants.

Using machine learning methods, we demonstrated the value of the gut microbiota composition in diagnosing extremely-low-birth-weight preterm infants. We assessed the clinical value of gut microbiota in ELBW preterm infants by a machine learning method, and found that the AUC values of the intestinal bacteria *Klebsiella*_ASV_2, *Enterococcus*_ASV_38, and *Klebsiella*_ASV_11 were 0.836. The AUC values for *Klebsiella*_ASV_2, *Enterococcus*_ASV_38, *Klebsiella*_ASV_11, *Acinetobacter*_ASV_51, and *Acinetobacter*_ASV_46 were found to be 0.793. The results show that the diagnosis of ELBW preterm infants based on gut bacteria is reliable, to some extent. With machine-learning analysis methods, gut bacteria may play a significant role in ELBW preterm infants, and their ROC values can predict diagnostic outcomes.

This study demonstrated a certain level of innovation: CST analysis is commonly used in the structural analysis of genital tract microbiota. In this study, we identified five major CSTs through an analysis of community types in low-birth-weight preterm infants, and CST was related to the clinical phenotype of premature infants. Furthermore, machine learning methods were employed to evaluate the potential of using bacteria composition in diagnosing preterm infants with ELBW. As for limitations, the 16S rRNA gene sequencing method used lacks the ability to analyze the functional composition of the gut microbiota. The study also lacked an independent validation cohort to verify the potential of using the bacteria composition in diagnosing preterm infants with ELBW. The next step will be to further investigate the functional aspects of the gut microbiota and conduct larger-scale validation studies.

## 5. Conclusions

The intestinal bacteria of premature infants are characterized by a community state type primarily driven by harmful bacteria such as *Enterococcus*, *Klebsiella*, and *Acinetobacter*. ELBW preterm infants exhibit an increase in the abundance of potentially harmful bacteria in the gut and a decrease in beneficial bacteria. These potentially harmful bacteria may be potential biomarkers for ELBW premature infants.

## Data Availability

The original contributions presented in the study are included in the article/supplementary material, further inquiries can be directed to the corresponding author/s.

## Supplementary Materials

The following supporting information can be downloaded at: https://www.mdpi.com/article/10.3390/children11070770/s1. Figure S1. Analysis of clinical phenotype diversity and network interaction visualization for preterm infants with low birth weight. (A) NMDS analyzes of differences in clinical phenotypes among the three groups of preterm infants; (B) A network interaction map of gut bacteria based on the clinical phenotypes of the infants.
