# Supplementary material for: The Association of Neonatal Gut Microbiota Community State Types with Birth Weight
Source: Children (Basel). 2024 Jun 25;11(7):770. doi: 10.3390/children11070770 (PMC11276374; doi:10.3390/children11070770)
Supplement: Supplementary file 1 [file children-11-00770-s001.zip › children-2997143-supplementary.pdf]

Supplementary Materials

1 Analysis of gut microbiota

The 16S rRNA gene sequencing

Firstly, the extracted fecal DNA was diluted to 1 ng/μl for use as template DNA. A polymerase chain reaction (PCR) was then performed using universal primers 515F (5'-GTG CCA GCM GCC GCG GTA A-3') and 806R (5'-GGA CTA CNN GGG TAT CTA AT-3'), which are specific to the V4 region of the bacterial 16S rRNA gene. PCR was performed using the Phusion® High-Fidelity PCR Kit (New England Biolabs, Ipswich, MA, USA) according to the manufacturer's instructions. Subsequently, the target bands were recovered using the QIAquick Gel Extraction Kit (Qiagen, Hilden, Germany). Library preparation was performed using the TruSeq® DNA PCR-Free Sample Preparation Kit (Illumina, San Diego, CA, USA). Finally, sequencing was conducted using the HiSeq2500 (Illumina, San Diego, CA, USA) in PE 250 bp mode.

Analysis of 16S rRNA gene information

Firstly, the paired-end reads were merged using FLASH software (version 1.2.7). Subsequently, quality control was performed using QIIME 2 software (version 2020.11) to obtain clean tags, and the clean tags were aligned with the Gold database (version 20110519). Chimera sequences were then detected using UCHIME software (version 7.0.1001). Non-chimera clean tags were clustered into operational taxonomic units (OTUs) with a similarity threshold of ≥ 97% using Uparse software (version 7.0.1001). Finally, Mothur software was used to align the OTUs with the SILVA database and perform species classification annotation. MUSCLE software (version 3.8.31) was employed to investigate the phylogenetic relationships between the OTUs and the SILVA database. Before analysis, the OTU reads were normalized based on the minimum sequencing depth among samples.

Analysis of NJ-related intestinal bacteria

To screen for significantly different taxonomic compositions between groups, taxonomic components with low abundance (average relative abundance less than 0.1%) and low coverage (not detected in more than 20% of the samples) were first filtered out. The DESeq2 method was used to screen for significantly different taxonomic compositions of intestinal microbiota between groups. The parameters set were a P-value of less than 0.05, a Benjamini

and Hochberg false discovery rate (FDR)-corrected P-value of less than 0.001, and an absolute value of log2 fold-change greater than 1.2.

ANOSIM analysis

Firstly, based on the genus-level data, the vegdist function from the R package Vegan was used to calculate the Bray–Curtis distance. Then, based on the obtained Bray–Curtis distances, the anosim function from the R package Vegan was further utilized to calculate the sample similarity matrix. Finally, the stat_boxplot function from the R package ggplot2 was used to perform and visualize the similarity comparison analysis within and between groups.

2 Random forest model

Firstly, using the train_test_split function from the model_selection module in the sklearn package (version 0.23.1) in Python (version 3.7.6), all samples were divided into training and testing datasets. The parameters were set as follows: test_size = 0.4, random_state = 2020. Then, the RandomForestClassifier from the ensemble module in the sklearn package was imported as the RandomForest model, and the parameters for the RandomForest model were set as follows: random_state = 0, n_estimators = 100, oob_score = True, n_jobs = −1. Based on the training dataset, the RandomForest model was fitted. The feature importances were sorted using the feature_importances_ function of the RandomForest model. The barh function from the pyplot module in the matplotlib package (version 3.2.2) in Python was then used to visualize the important features. Finally, the roc_curve and auc functions from the metrics module in the sklearn package were used to conduct ROC analysis and evaluate the AUC score for the test samples, respectively. Visualization was then carried out using the plot module from the matplotlib package. To obtain the 95% confidence interval for the AUC score on the test samples, the cross_val_score function from the model_selection module in the sklearn package was used to perform cross-validation analysis with the parameter cv = 3. The 95% confidence intervals were calculated using the formula: scores_cv.mean() ± scores_cv.std() × 1.96.

3 Supplementary Figure

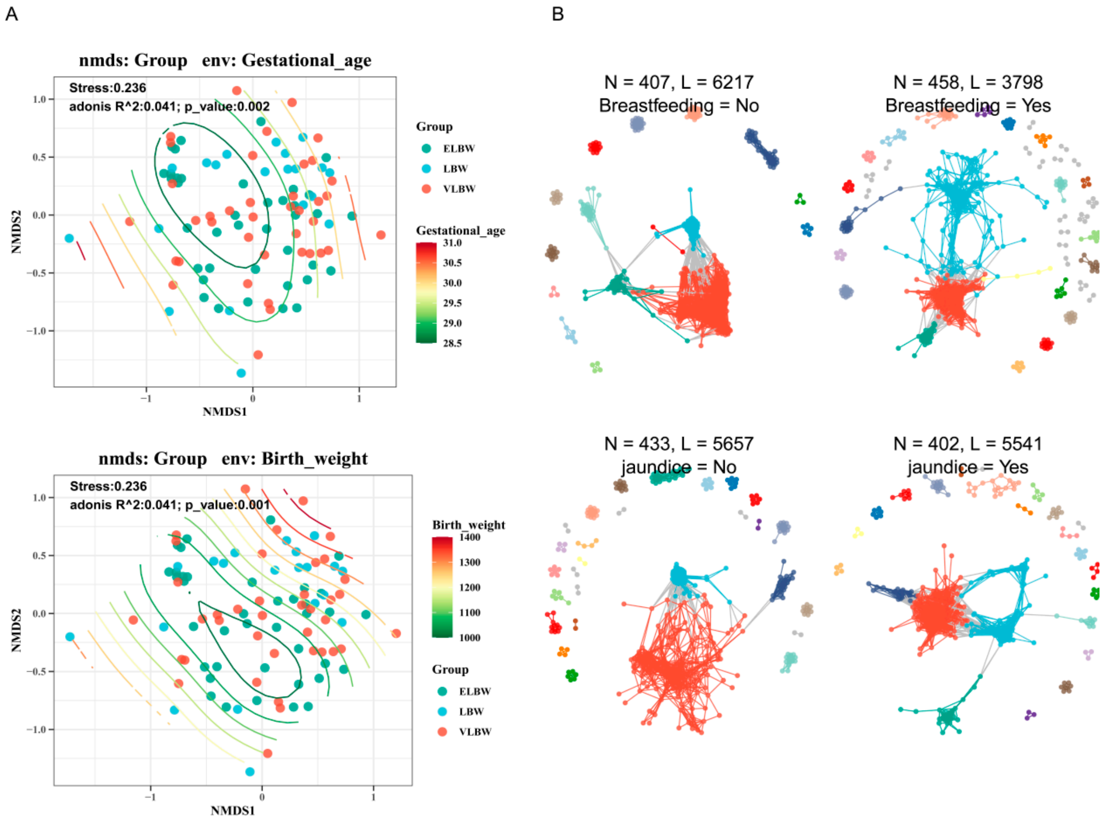

Figure S1. Analysis of clinical phenotype diversity and network interaction visualization for preterm infants with low birth weight. (A) NMDS analyzes of differences in clinical phenotypes among the three groups of preterm infants; (B) A network interaction map of gut bacteria based on the clinical phenotypes of the infants.
