# Supplementary figures and images for: The Association of Neonatal Gut Microbiota Community State Types with Birth Weight
Source: Children (Basel). 2024 Jun 25;11(7):770. doi: 10.3390/children11070770 (PMC11276374; doi:10.3390/children11070770)

A

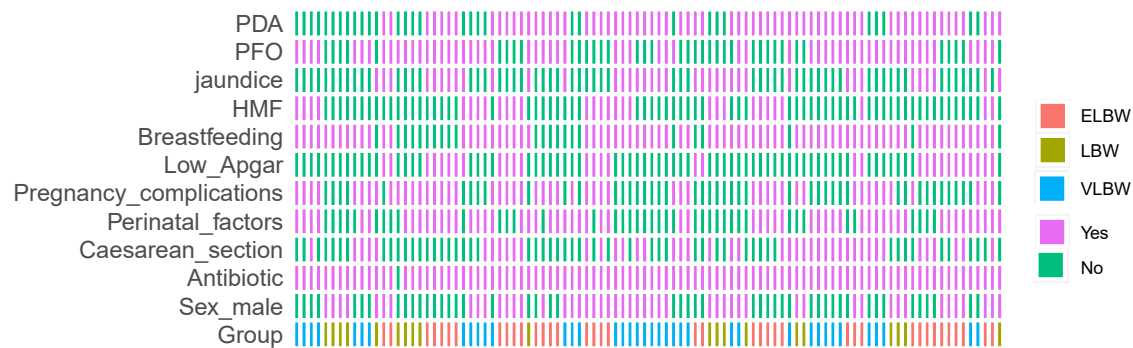

B

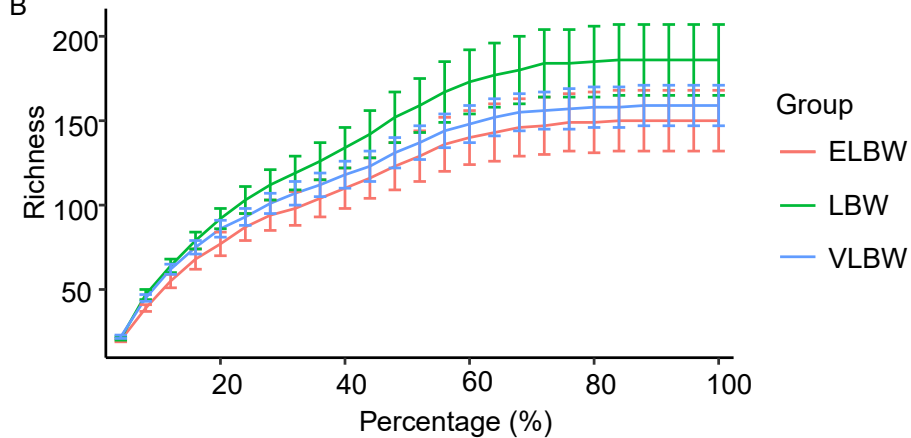

C

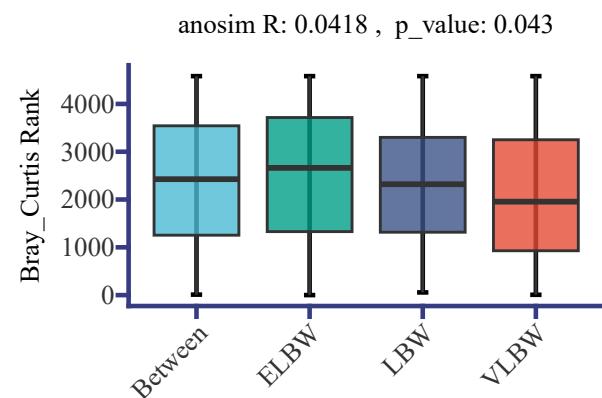

D

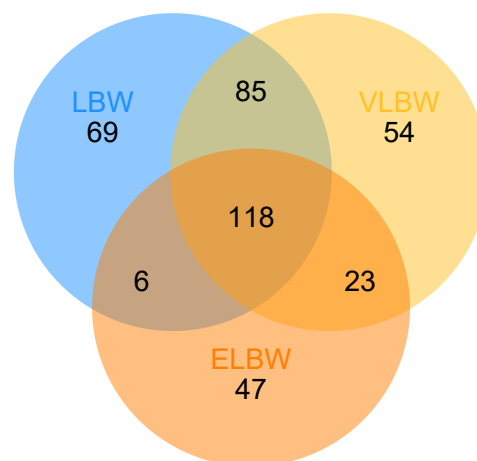

E

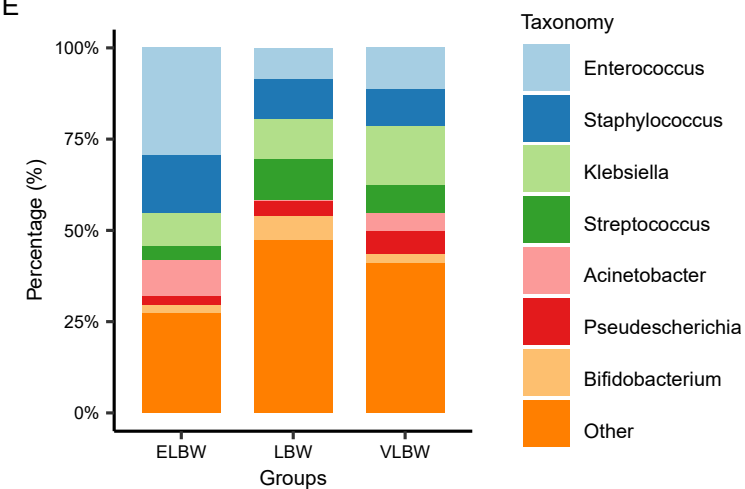

F

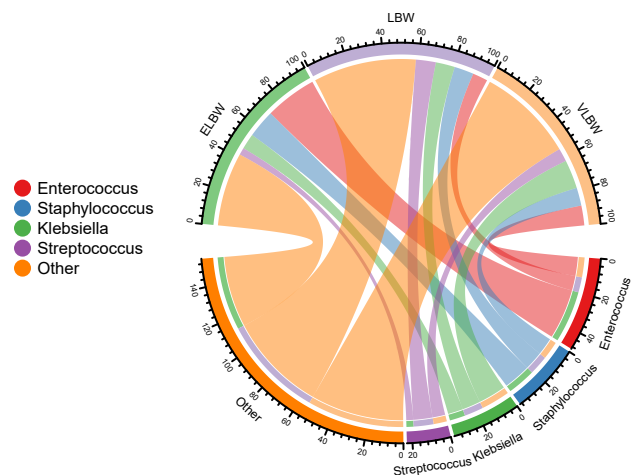

G

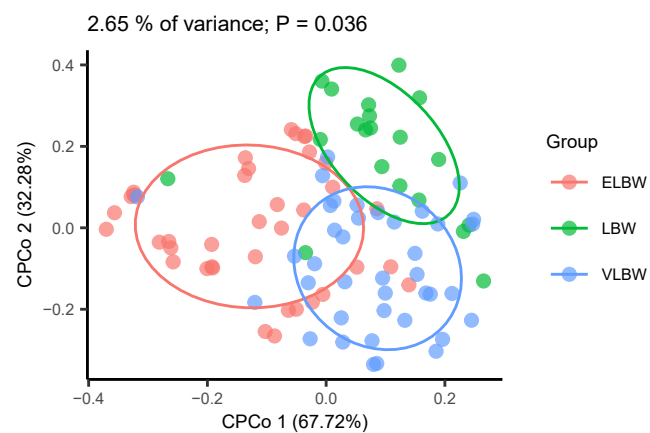

H

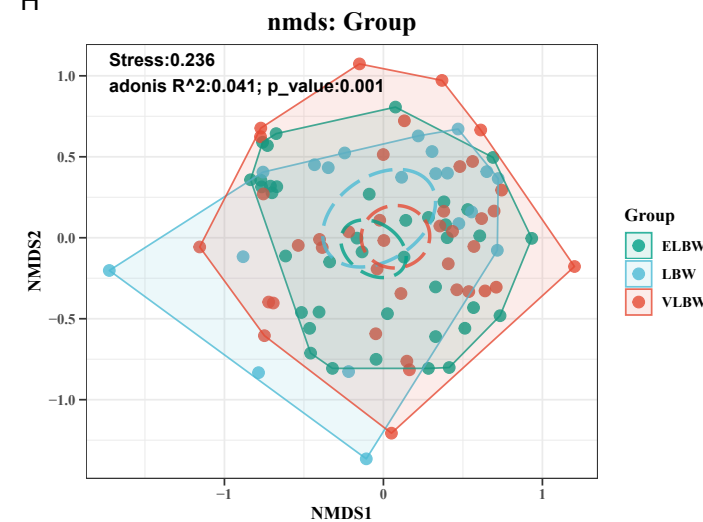

Supplement: Supplementary file 1 [file children-11-00770-s001.zip › Figure legends/Figure 1.pdf]

A

ELBW-LBW

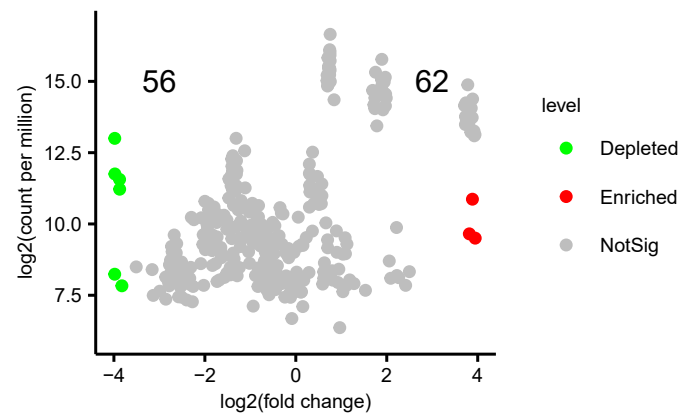

B

ELBW-VLBW

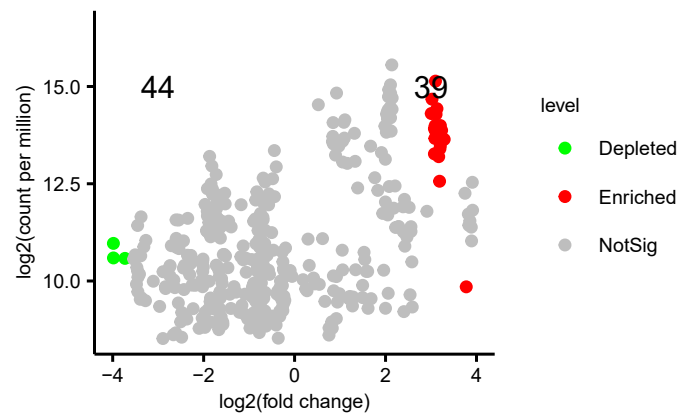

C

VLBW-LBW

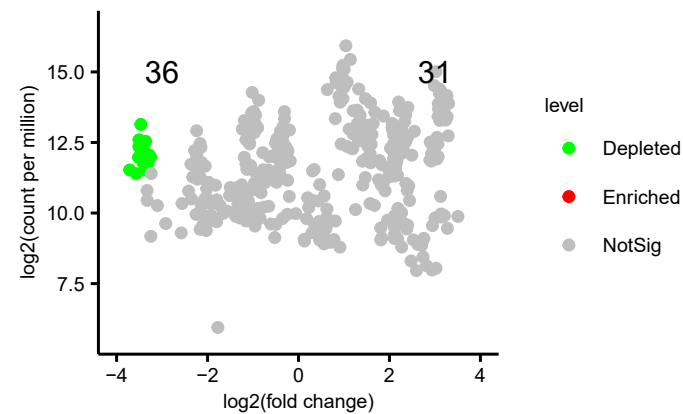

D

ELBW-LBW

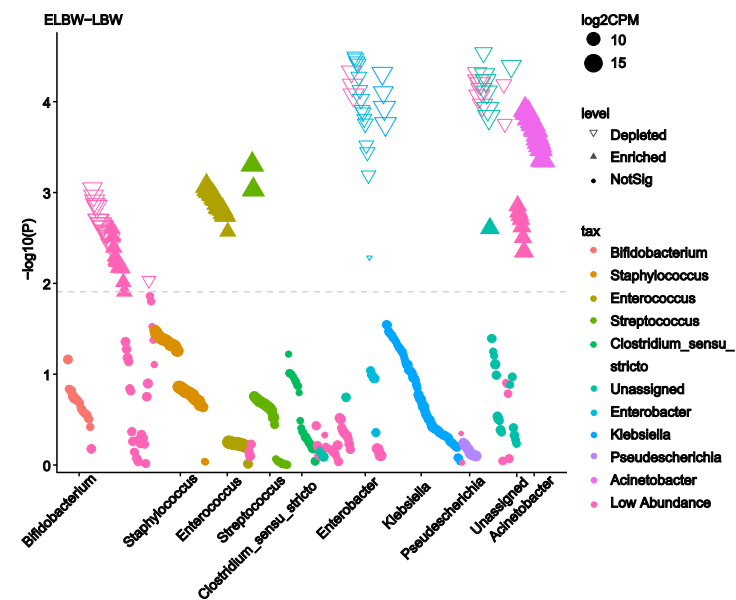

E

ELBW-VLBW

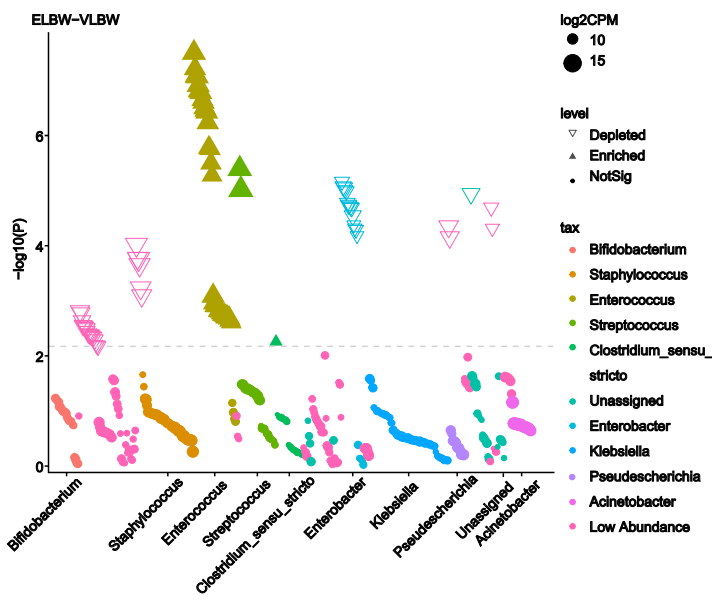

F

VLBW-LBW

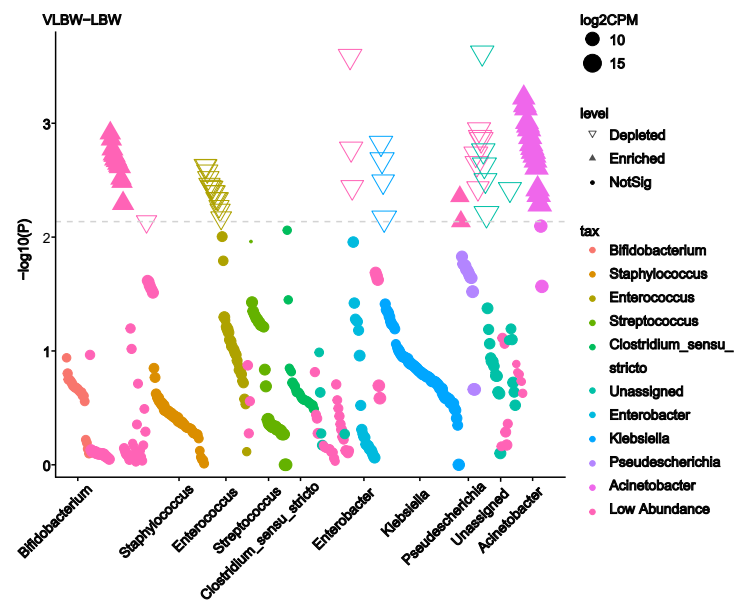

Supplement: Supplementary file 1 [file children-11-00770-s001.zip › Figure legends/Figure 2.pdf]

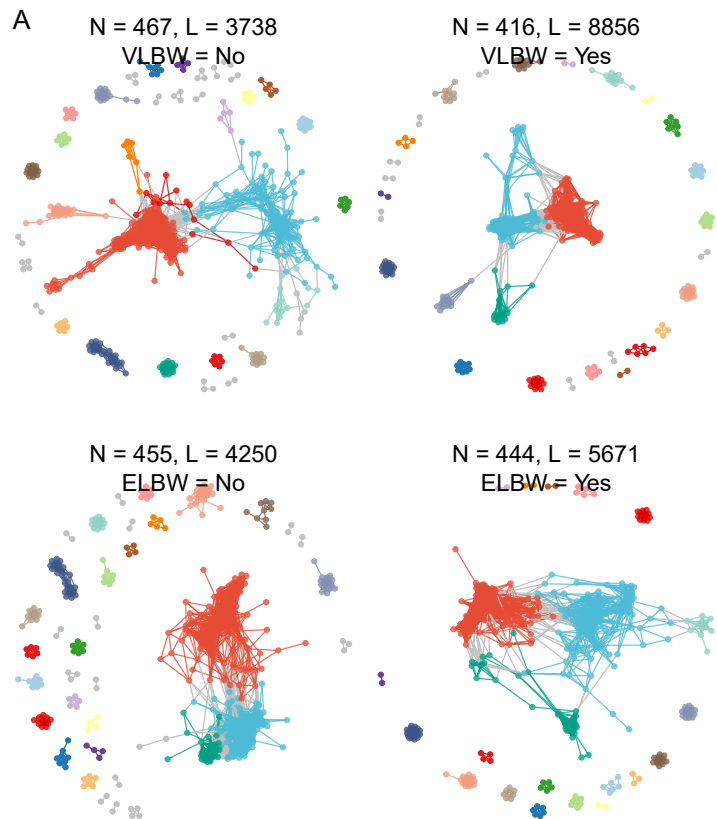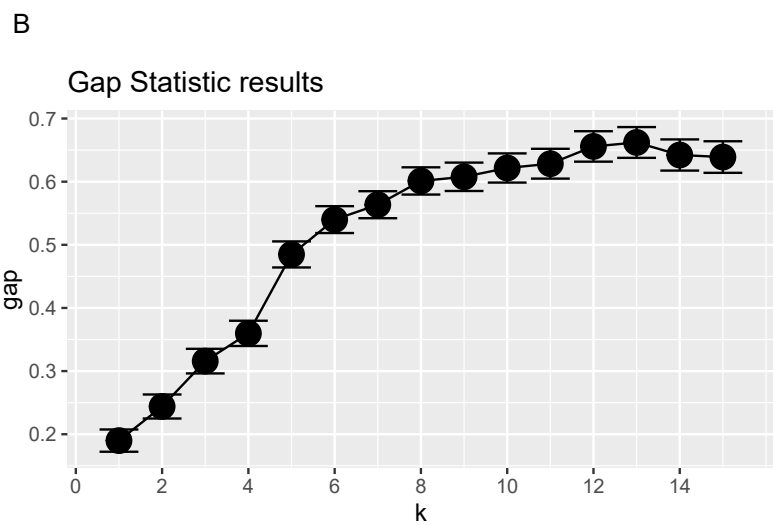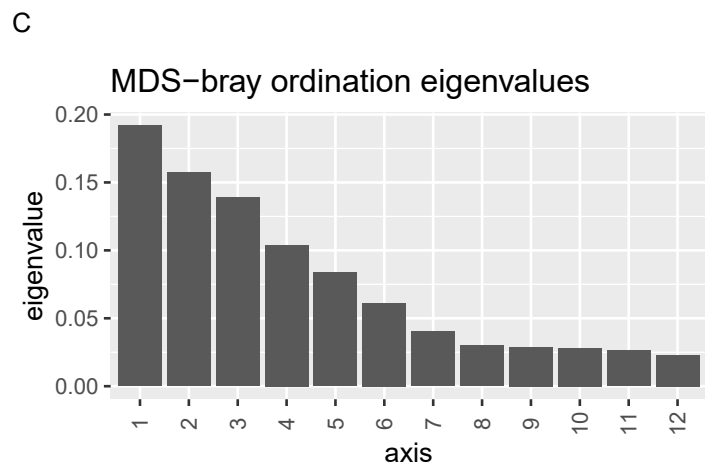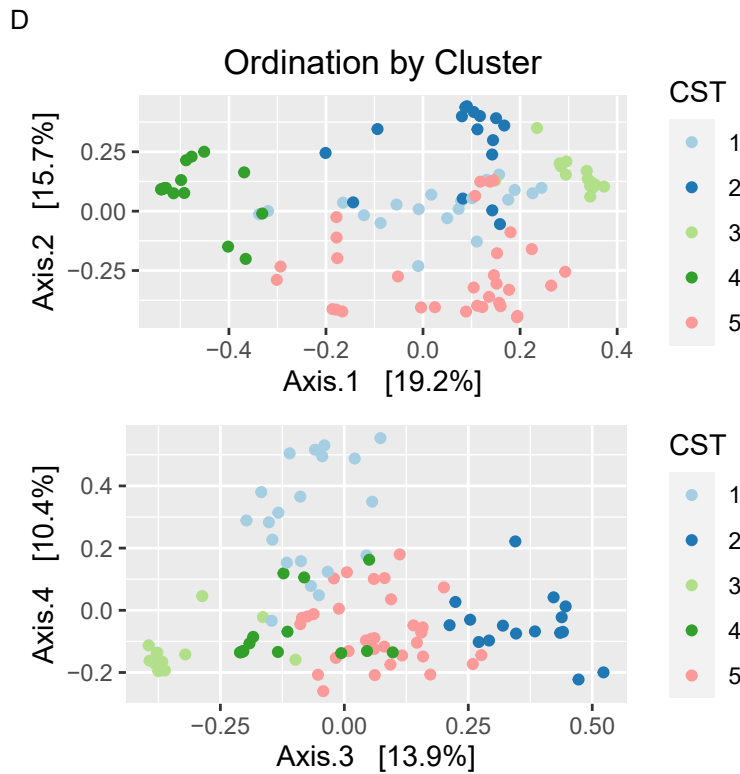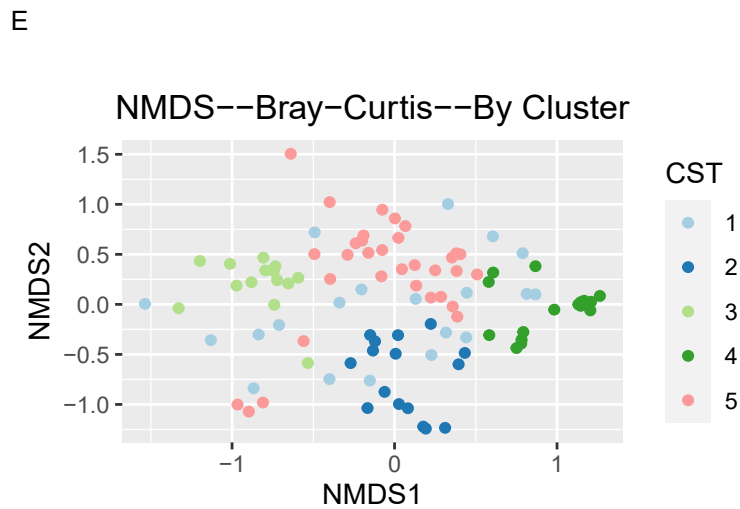

**F**

Supplement: Supplementary file 1 [file children-11-00770-s001.zip › Figure legends/Figure 3.pdf]

A

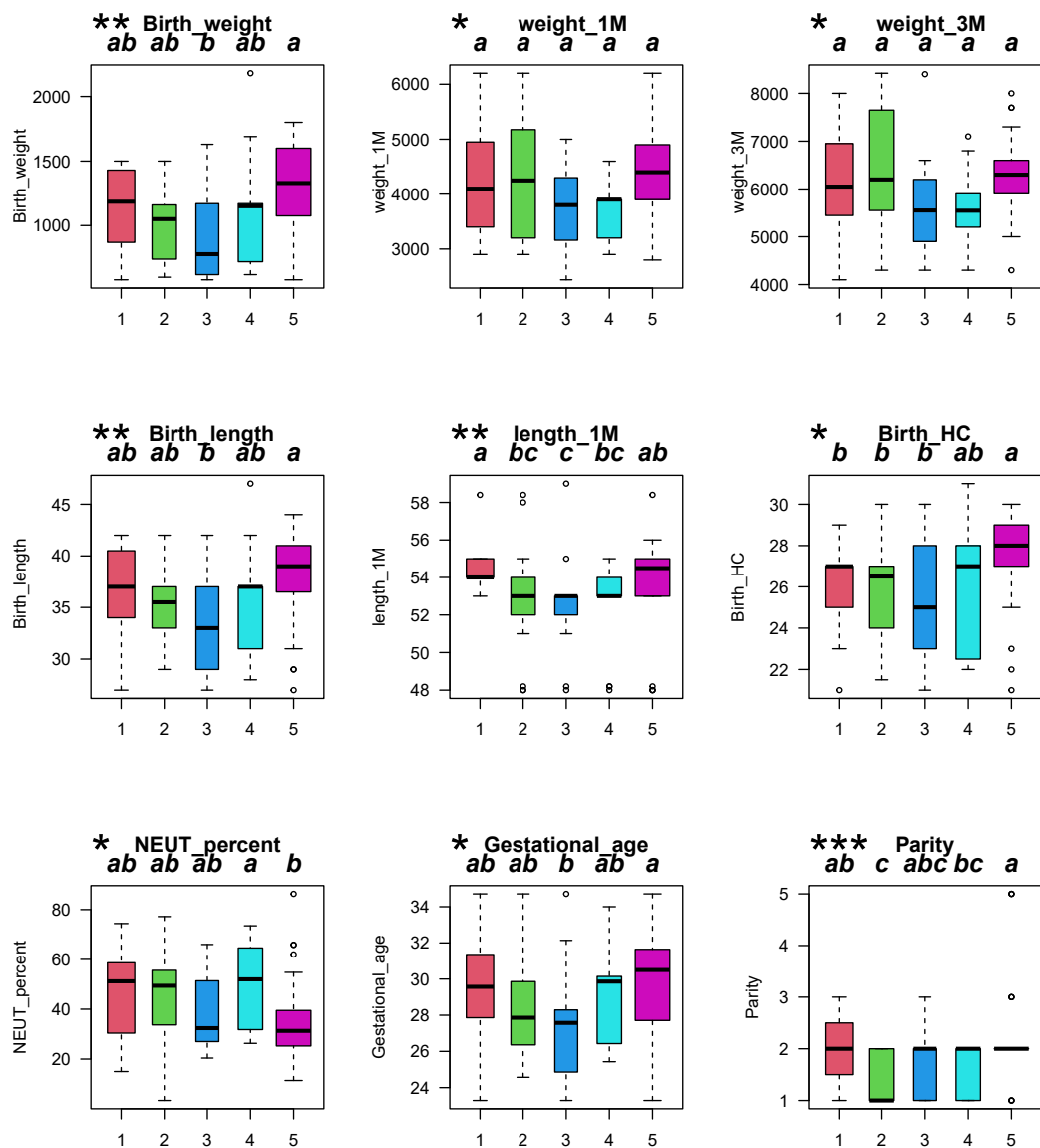

B

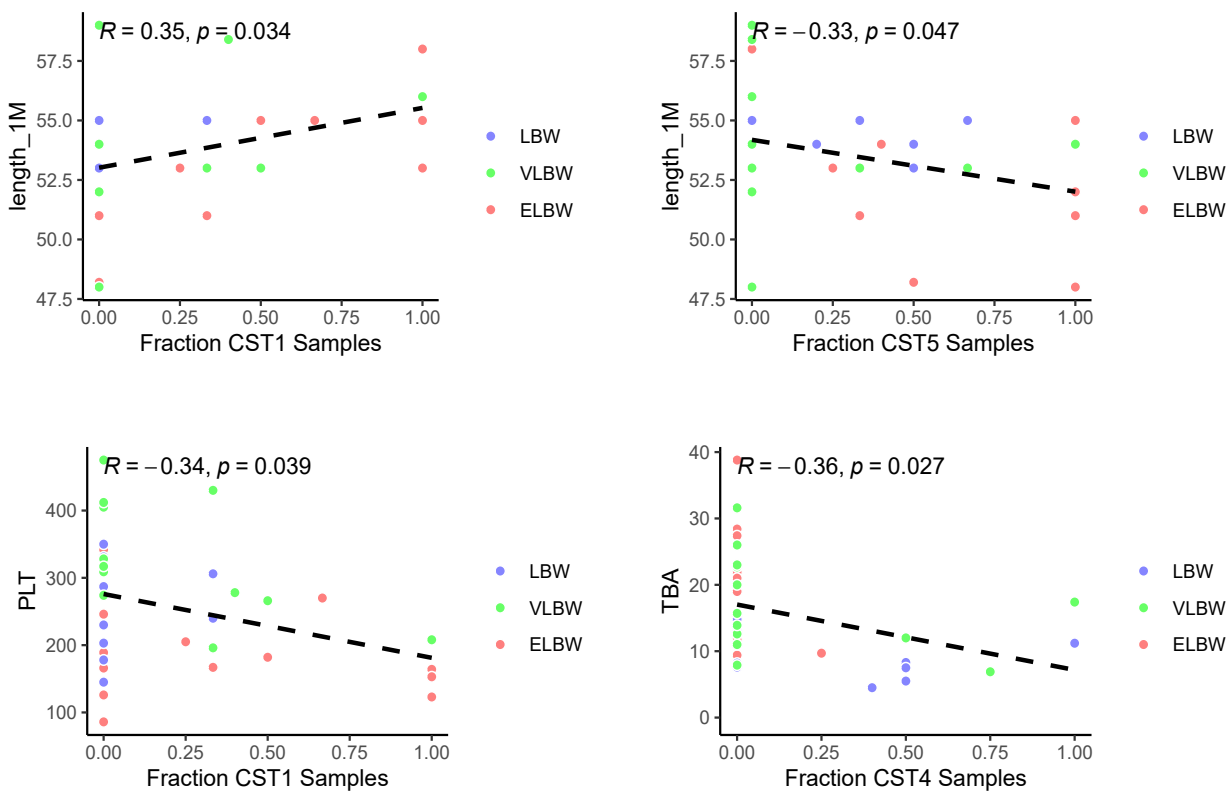

Supplement: Supplementary file 1 [file children-11-00770-s001.zip › Figure legends/Figure 4.pdf]

A

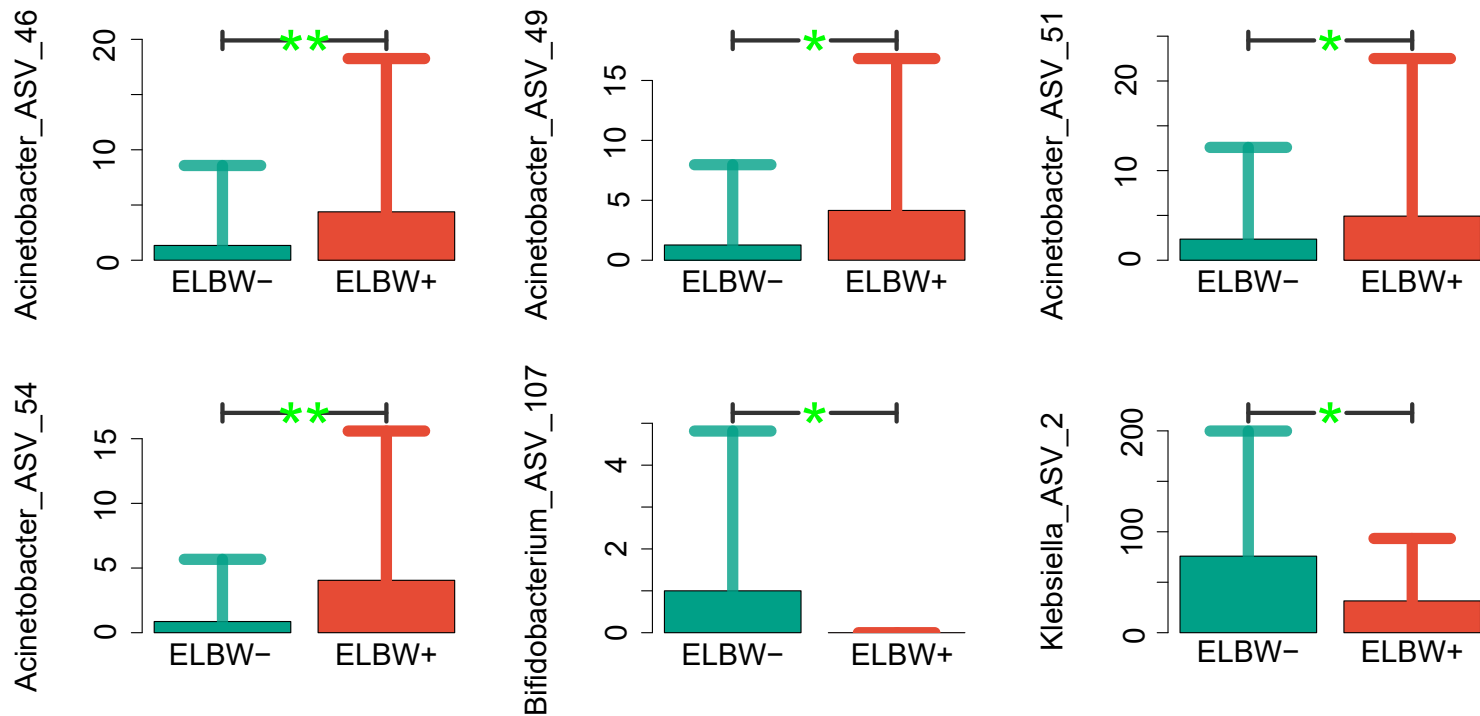

B

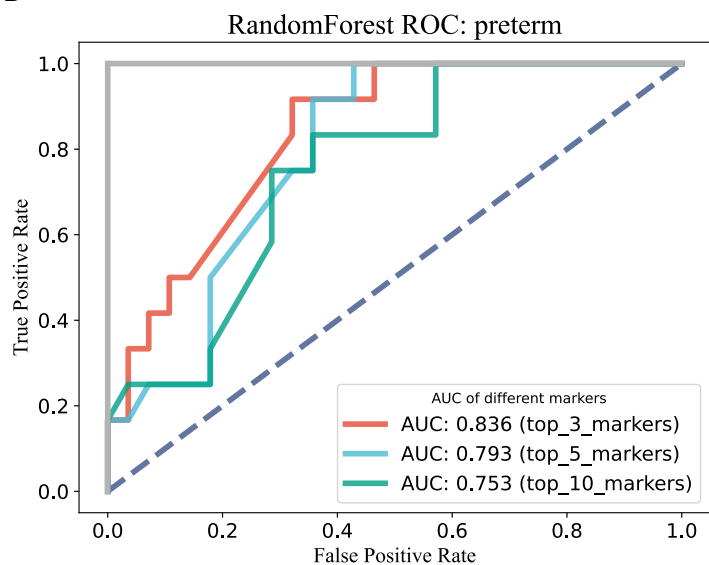

C

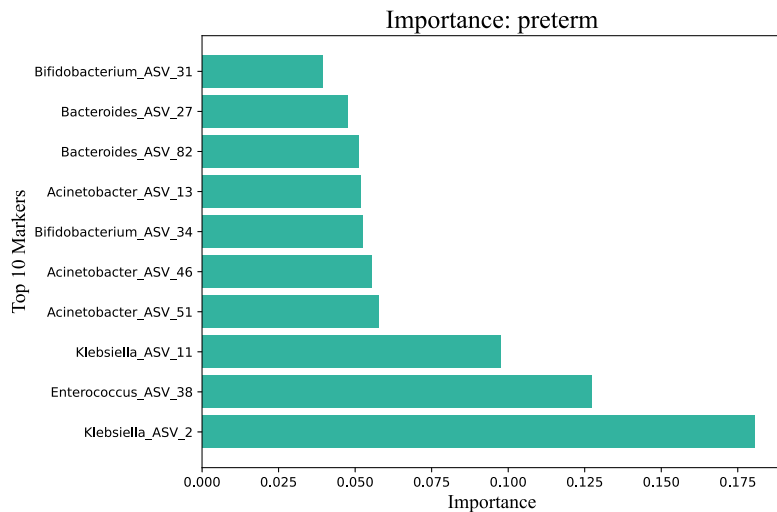

Supplement: Supplementary file 1 [file children-11-00770-s001.zip › Figure legends/Figure 5.pdf]

A

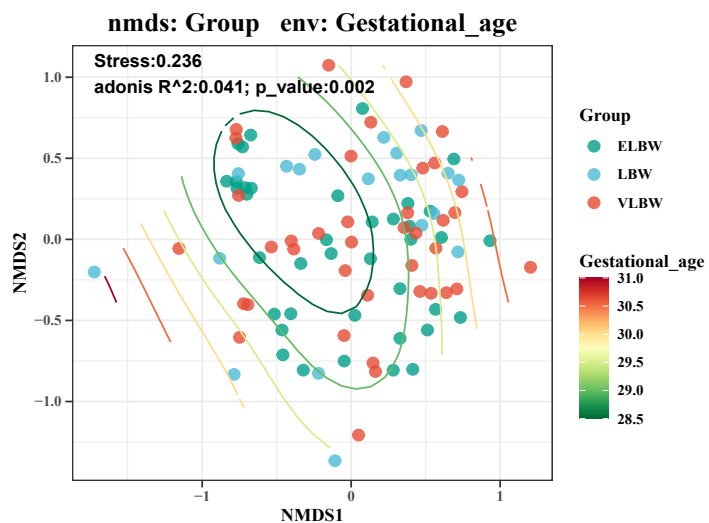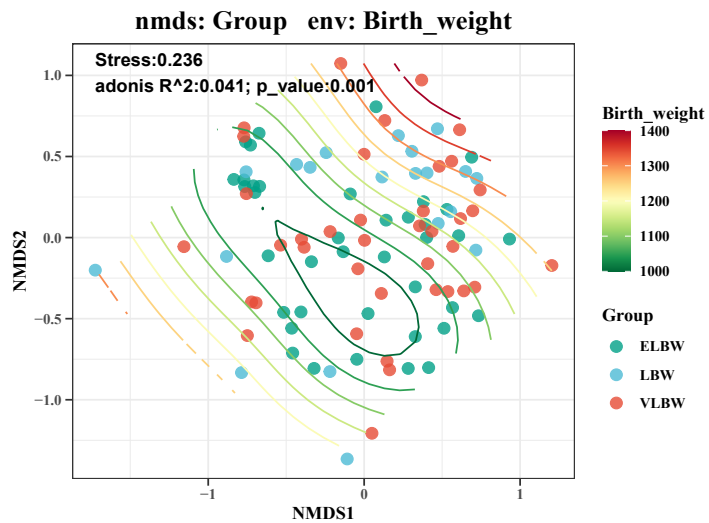

B

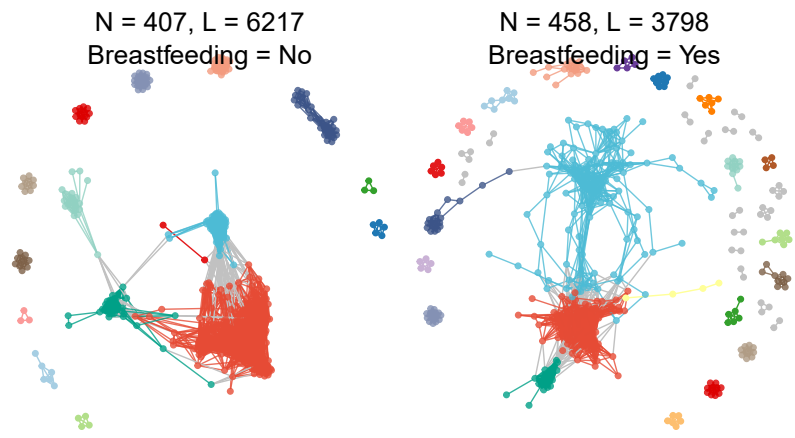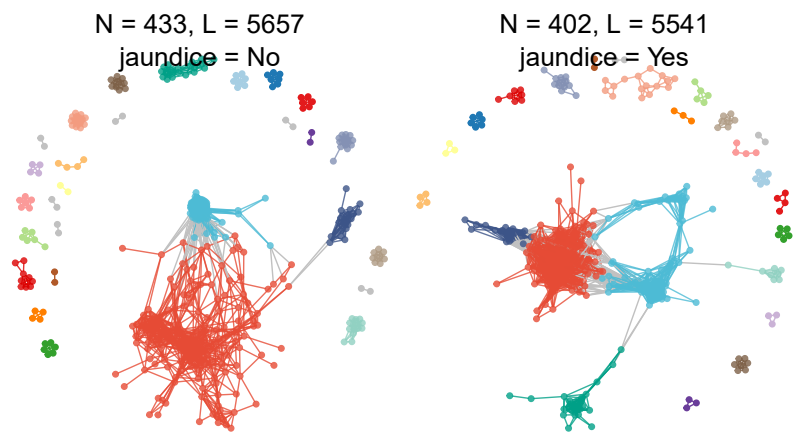

Supplement: Supplementary file 1 [file children-11-00770-s001.zip › Figure legends/Figure S1.pdf]
